# Design and Implementation of a Smart LED Lighting System Using a Self Adaptive Weighted Data Fusion Algorithm

**DOI:** 10.3390/s131216915

**Published:** 2013-12-06

**Authors:** Wen-Tsai Sung, Jia-Syun Lin

**Affiliations:** Department of Electrical Engineering, National Chin-Yi University of Technology, Taiwan No.57, Sec. 2, Zhongshan Rd., Taiping Dist., Taichung 41170, Taiwan; E-Mail: max79411@gmail.com

**Keywords:** intelligent LED, lighting system, self-adaptive weighted, data fusion, ZigBee, IR

## Abstract

This work aims to develop a smart LED lighting system, which is remotely controlled by Android apps via handheld devices, e.g., smartphones, tablets, and so forth. The status of energy use is reflected by readings displayed on a handheld device, and it is treated as a criterion in the lighting mode design of a system. A multimeter, a wireless light dimmer, an IR learning remote module, *etc.* are connected to a server by means of RS 232/485 and a human computer interface on a touch screen. The wireless data communication is designed to operate in compliance with the ZigBee standard, and signal processing on sensed data is made through a self adaptive weighted data fusion algorithm. A low variation in data fusion together with a high stability is experimentally demonstrated in this work. The wireless light dimmer as well as the IR learning remote module can be instructed directly by command given on the human computer interface, and the reading on a multimeter can be displayed thereon via the server. This proposed smart LED lighting system can be remotely controlled and self learning mode can be enabled by a single handheld device via WiFi transmission. Hence, this proposal is validated as an approach to power monitoring for home appliances, and is demonstrated as a digital home network in consideration of energy efficiency.

## 1. Introduction

As an increasingly popular issue, the field of digital home services appeals to plenty of high tech companies. The way humans go through their daily lives in today's Hollywood films could be realized in the very near future, one of which is the digital home network aimed at facilitating human's daily lives. Currently, digital home network technology is being developed with focus on six aspects, namely, central control systems, security monitoring, heath care, residence monitoring, information appliances, and energy saving. The field of central control covers system control, management authority, *etc.* security monitoring covers environment monitoring, building access control, *etc.* health care covers patient location tracking, bed management in hospitals, *etc.* residence monitoring covers lighting control, *etc.* information appliances cover home automation control, and energy saving covers efficiency improvement, power management, *etc.* Currently, many companies have put a great effort into the development of central control and information appliances, while they do not pay as much attention to the field of health care. This study is devoted to the applications of residence monitoring and information appliances.

There exists a wide diversity of home electronics with incompatible remote controls. The motivation of this work is hence to develop a platform, either on a smart phone or a tablet, for interoperability among these incompatible remote controls, such that the real time monitoring on home energy use can be achieved, and the brightness as well as the lighting modes of a smart LED lighting system can be switched. Smart control refers to a succession of control strategies, involving experience learning, logic operation, adaptivity, organization, debug, and so on, and is widely applied to highly uncertain, nonlinear, or complicated systems, which cannot be well controlled by conventional approaches.

**Figure 1.**
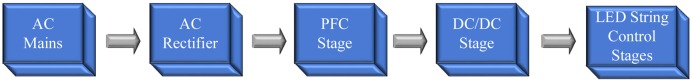
Flow chart of a smart LED lighting system.

A clear disadvantage of a conventional lighting system is that it lacks the flexibility for any relocation of light sources, and it requires a great effort to rewire the entire system once it gets big, e.g., in a high-rise office building, *etc.* These days, the instant energy use in lighting in such a high-rise building must be monitored in real time for energy saving purposes. A smart lighting system refers to an MCU-based system integrating automation, electronics, computer, network communication, and many more for energy efficiency improvement. In a conventional lighting system, a light source can be merely switched on/off manually, while, instead in a smart one, various preset lighting modes are preloaded into the lighting system, either wired or wireless, to meet the user's specific needs. Besides, conventionally, a heavily loaded lighting system necessitates a high-capacity switch, and requires a large volume of cables to drive a distant load. In contrast, a load is directly powered by an output driver, meaning that there is no need to increase the power capacity of a switch when the system is heavily loaded, and it merely requires a long signal line to drive a distant load. Furthermore, a smart lighting system can be made dimmable and controllable by timer means. As illustrated in Figure 1, a smart LED lighting system comprises a rectifier followed by a power factor corrector and then by a DC/DC converter [1].

As a rule, there are two approaches to energy efficient lighting, namely, the use of high efficiency light sources, and the development of smart lighting techniques. An illustration of the latter is the thermal infrared sensing technique, by use of which indoor lights can be switched on/off automatically when there is somebody/nobody present. On top of that, a lighting system can be made adaptive, such that the indoor brightness can be maintained at a constant level taking into account the contribution of outdoor sunshine. As indicated by statistics, lighting, air conditioning and the rest account for 33%, 50% and 17% of energy consumption, respectively. Since the late 1960s and early 1970s, developed countries started to develop green lighting technologies for ecological concerns.

A great challenge to be faced is the electrical wiring problem when try to build an energy efficient lighting system in an old building. Is there a way to get the job done, but not to rewire the whole house? The answer is affirmative. A solution to this problem is the use of short range wireless communication techniques, namely, Bluetooth, IEEE 802.11 WiFi and infrared. For instance, the residence lighting can be controlled by an IR remote control. There are multiple remote controls in most residences, and a universal remote control is a must such that any of the home appliances can be controlled by such single piece [2].

**Figure 2.**
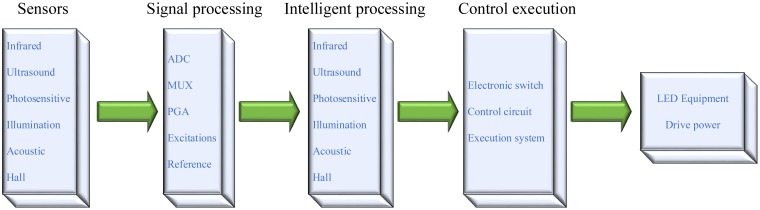
Operation sequences of a smart LED lighting system.

A wide variety of sensors, including IR, ultrasonic, light, illumination, voice, and Hall sensors, can be integrated into an MCU-based LED lighting system. In this manner, various types of detected signals can be processed in such a way that an LED lighting system can be operated in a smart way. Due to the very weak sensed signals, the front end electronics and signal processing is seen as required and involves an ADC(s), an MUX, a PGA, a voltage reference, an excitation source, embedded micro processor (MCU, ASIC), memory (RAM, E2PROM), *etc.* Smart signal processing can be automatically performed on the collected data by either an MCU or an ASIC inside commercial products. As illustrated in Figure 2, a number of wireless communication modules, including ZigBee, WLAN, TCP/IP, WiFi, *etc.* can be integrated into a remote control smart LED lighting system.

## 2. Literature Review

As surveyed in [3], current digital home applications mainly cover six aspects, *i.e.*, central control systems, security monitoring, heath care, residence monitoring, information appliances, energy saving. A central control system can be applied to fridge monitoring, and remote control living room, the security monitoring technique is applied to garage door control, car anti-theft devices, gas leakage monitoring, shower temperature monitoring, building access control, emergence call system, fire alarm, video surveillance systems, and many more. Conventionally, the performance of a security monitoring system has a direct relationship with the number of security cameras installed, and there are inevitably some blind points when videotaping. A long term video monitoring brings about a multitude of audio and image data. As suggested in [4], a novel security monitoring system is operated in such a way that a residence owner can be warned by a text alert automatically, and a motion activated security camera(s) is operated only when there is something wrong therein for energy saving purposes.

Health care technology is applied to smart beds, electric adjustable beds, smart toilets, smart sofas, smart first aid kits, *etc.* residence monitoring technology is applied to smart closets, smart lamps, electric curtain control, automated temperature monitoring, *etc.* information appliance control is applied to smart kitchens, digital TVs, image phones, *etc.* and energy saving technology is applied to energy saving microwave ovens, electric ovens, energy efficient wastewater treatment, energy saving heaters, thermal bathtubs, *etc.* As indicated in [5], facial feature extraction is performed before family members return home, and the images of unidentified visitors can be displayed on monitors or smart phones for security concerns. Besides, the region of interest within an image can be skillfully specified in an attempt to reduce the incidences of false alarms. This technology can be further applied as an auxiliary tool for parents to keep an eye on infants and children. As stated in [6,7], optical fiber technology has been demonstrated as a very effective approach to the integration of smart TVs and many other services. For instance, at a minimum transmission rate of 100 Mbps for home use, high speed network services, including high definition TV (HDTV), video on demand (VOD), and the like, can be realized. Any type of optical fiber wireless products is not available yet in market due to a lack of transmission protocol stipulated. Other than the high spending, it requires a great effort to implement fiber to the home projects. Currently, there have been a wide variety of technologies, including LED, CCD and CMOS sensor technology, solar energy technology, available for digital home services.

There are a great number of problems waiting to be solved when integrating a wide diversity of digital home services. As suggested in [8,9], a great challenge encountered is the platforms and interoperability among various technologies, such as Ethernet, phone lines, power lines, IEEE 1394, USB2.0, Bluetooth, infrared, 802.11a/b/g, and so forth. Another big challenge is to provide the required network flexibility such that any extra sensors or devices can be effortlessly introduced into an existing smart home service network. As pointed out in [9], another challenge is to find effective ways to make new technologies accessible for seniors.

In this work, the addition of extra IR remote control home appliances into a digital home network merely requires more IR output channels in the aspect of hardware, while additional control interfaces must be developed in the aspect of software instead. As indicated in [1], an MCU-based LED lighting system is operated in such a way that the aim of cost reductions and an energy saving plan can be implemented. In an effort to extend LED lifetime, an LED light source is dimmed or even switched off once the sensed operating temperature goes beyond a threshold, and is lit in a dark place. Furthermore, a greater number of street lights can be lit automatically in rush hour traffic due to safety concern, and adaptive brightness is enabled according to the number of people indoor. A smart LED lighting system is proposed based on [1] toward the energy consumption reduction target.

If the LED heat has no way to exit the LED would be continuously enveloped in high temperatures. After a period of time the heat will cause the LED to become unstable with a decrease in brightness. Until now most LED lights were used with cooling aluminum housing material. There are three types of aluminum heat sinks, die-cast aluminum, extruded aluminum and fin-style aluminum. They have different degrees of cooling capacity, of course, the cost is different and so the LED lamp is priced differently. Therefore, when you choose LED lights you should try to understand its thermal material, as even with a low voltage LED chip heat will be released. LED heat radiating is not good in that it reduces the LED lights life-time to only 8,000–15,000 h or less. This is the most important factor causing increased costs.

## 3. Design of an LED Lighting System

All the constraints must be taken into account, e.g., the operating conditions, limitations on electrical and optical components, cost, LED driver current, and expected system lifetime, when an LED lighting system is designed so as to meet the user's specific needs, e.g., temperature ratings, expected brightness.

**Figure 3.**
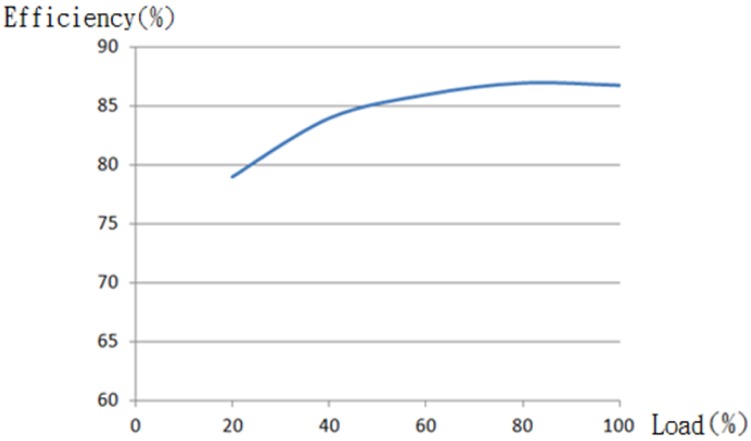
A plot of the LED driver efficiency *versus* load.

There is no way that an LED lamp can be made 100% efficient due to the inevitable power loss in the driver of the lamp. For this sake, the power loss must be taken into account in the design phase of an LED lighting system. Typically, an LED driver is measured to have an efficiency ranging from 80% to 90%, while an LED driver with a high efficiency over 90% is a high price one. As illustrated in Figure 3, the driver's efficiency is found as a function of load. It is noted that a load above 50% is recommended for optimized efficiency, namely minimized cost. For indoor use, an efficiency of 87% is highly recommended, while a lower one is recommended for outdoor use or for an extended lifetime.

The power loss leads to a drop in the lamp efficiency, and the number of LED lamps is determined so as to meet the total luminous flux requirement, expressed as:

(1) $$B_{s}=B_{M}/(\eta_{G}\eta_{R})$$

where *B_s_* and *B_M_* represent the actual luminous flux and the target value, respectively, *η_G_* and *η_R_* the optical and the thermal efficiencies, respectively.

LED operating current plays a critical role in the lighting efficiency and the lifetime thereof. A rise in the operating current brings about an enhanced output power and requires a smaller number of LEDs, but the price paid is a degraded efficiency, a larger sized LED driver and a shorter life cycle, since there is a high temperature drop across LED heat channels. The minimum, rather than typical, luminous flux requirement, as specified in an LED application note, must be met in the determination of the number of LED lamps. Accordingly, the number of LEDs, *S_LED_*, is given as:

(2) $$S_{\text{LED}}=B_{S}/B_{D}$$

where *B_S_* denotes the actual luminous flux, and *B_D_* the minimum flux emanating from each LED. Since each LED is an independent light source, an LED lamp provides a much longer life cycle than a conventional light source, and can be integrated with novel light sources into an existing lighting system.

Although LED is an energy efficient light source, a large amount of heat consumption is generated in an LED lighting system when a great number of high power LEDs operate concurrently. The efficiency of an LED is found to decrease with the operating current, namely, the operating temperature. In an effort to operate an LED at a high efficiency, the operating temperature must be kept low for sure. A significant heat dissipation improvement has been made using a heat sink to which a high power LED is mounted. In contrast, the outward transfer heat generated by an LED lamp cannot be made as efficient as in the preceding case, due to the thermally non conductive material used in a lamp case. In this context, a steep temperature rise brings about a degraded performance and a shortened lifetime. There is a great challenge when dealing with a theoretical thermal analysis on an LED lamp due to the thermal convection with complicated boundary conditions and the thermal conduction across multiple interfaces. As a matter of fact, there is no need to analyze the heat distribution in a non-equilibrium state, since merely an equilibrium state is the issue of interest. It is an extremely difficult task to evaluate the heat distribution in the interior of a lamp case across multiple interfaces, and what really matters in practical application is whether the temperature falls below the rating. A solution to this problem is the use of thermal resistance theory, an advantage over streamline thermal analysis in this case.

There is a huge difference between the operations of an LED, an incandescent lamp and a fluorescent lamp. Both an incandescent lamp and a fluorescent lamp are devices powered by AC 220 V, albeit a fluorescent lamp requires a rectifier circuit and a switch. Yet, an LED lamp is indeed a DC operated device, meaning that AC 220 V must be rectified into DC in advance. A low efficient LED driver will degrade the total efficiency of the lamp, according to which a way must be found to elevate the driver's efficiency. In most cases, there are two ways to tune LED intensity, one of which is achieved by the change of DC driving current, and the other is made by pulse width modulation (PWM). The light intensity varies linearly with the driving current, until a threshold is reached. Light efficiency degradation is seen together with a large amount of heat at high driving currents. The light intensity is tuned according to the Talbot-Plateau law in a PWM scheme. Commercial LEDs are available with recommended driver circuits meeting EMI and other safety requirements, leading to a short design phase. Yet, there exists a problem that the drivers recommended are measured as a rule to demonstrate an efficiency of approximately 80%, and driver's performance variation, affecting the life cycle together with the operating temperature of an LED, appears among those provided by LED manufacturers [10,11].

## 4. System Frame

As a server, XP-8000 takes charge of data access, and gets connected to a Touch Pad, a multimeter, an IR learning remote module, a wireless lighting controller, and many more through an RS-232 or an RS-485 interface such that a digit home is constructed.

**Figure 4.**
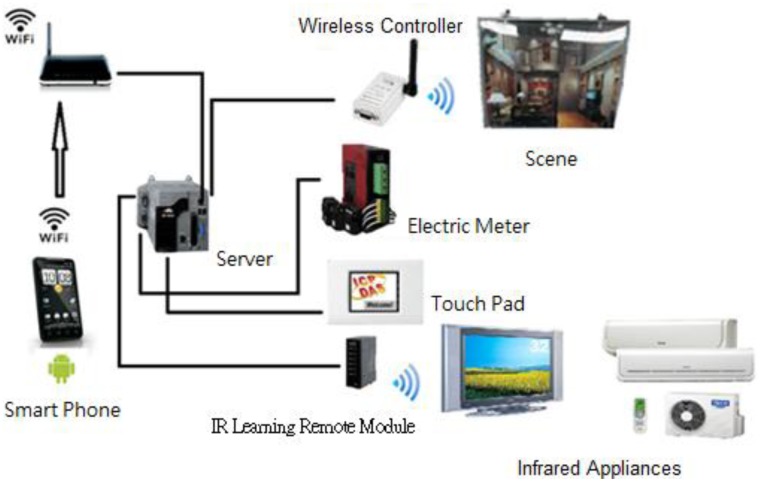
Devices controlled by the server in a smart lighting system.

As illustrated in Figure 4, an IR learning remote module is wired to the server, an XP-8000, through an RS-232 interface, while the voltage/current readings on a multimeter is transmitted to the server by means of an RS-485 interface. A light module is instructed via a wireless controller connected to and by the server through the RS-232 interface, and the server is operated according to the command issued by a Touch Pad through WiFi connection.

In the system server, a particular block of memory, referred to as the Shared Memory hereafter, is reserved for the current status storage of various electrical devices. For instance, instructions are issued indirectly from a mobile device, e.g., a smart phone or a tablet, using WiFi technique by way of the Shared Memory. In contrast, devices are instructed directly by a human computer interface on a touch screen, subsequent to which the status information stored in the Shared Memory is updated. Accordingly, the readings displayed on a multimeter can be presented on a smart phone, a tablet or a human computer interface on a touch screen by means of the Shared Memory.

### 4.1. XP-8000 Controller as a Server

**Figure 5.**
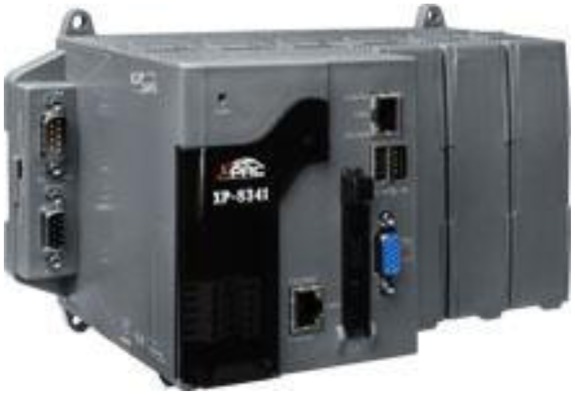
Photo of an XP-8000 controller as a server.

As a server in this proposal, XP-8000 is a new generation of programmable automation controller. Equipped with AMD Geode LX800 500 MHz processor, it is loaded with Microsoft Windows Embedded Standard 2009 as its operating system, featuring USB interfaces, Ethernet, RS-232/RS-485 channels and a VGA port. XP-800 series is designed to provide 3–7 expansion slots, into which high performance parallel/serial I/O modules can be plugged, as illustrated in Figure 5.

The XP-8000 is designed to take the readings on a multimeter using elogger, and issue command to an IR or a light control module using C programs.

### 4.2. Touch Pad Touch Pad as Human Computer Interface

**Figure 6.**
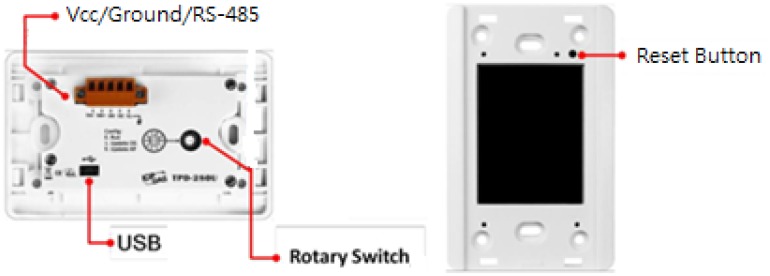
Back and front panels of a Touch Pad.

The touch pad is employed as a human computer interface on a touch screen for lighting mode selection, light tuning, and the consumer electronics control, and signal reading from a multimeter, as illustrated in Figure 6.

### 4.3. PM-213x PM-213x Digital Multimeter

**Figure 7.**
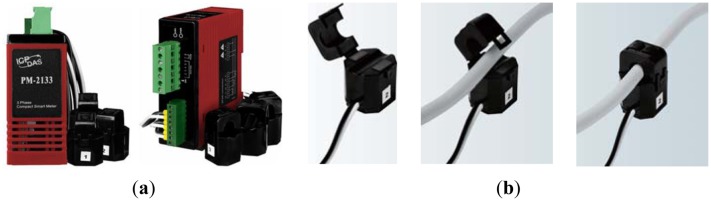
Photos of (**a**) a multimeter and (**b**) a clamp-on current transformer.

With a wide measurement range, PM-213X series is widely applied to measurement and monitoring of regular single and tree phase power systems. As illustrated in Figure 7, it features a clamp-on current transformer (CT), long term monitoring, standard communication interface, small size, easy installation, low cost and high flexibility. With a measurement range of 60 A (Φ10), extensible into 100 A (Φ16) or even 200 A (Φ24), it has been widely applied into the monitoring of building power systems and plant facilities.

### 4.4. IR-210–Universal Infrared Learning Remote Module

**Figure 8.**
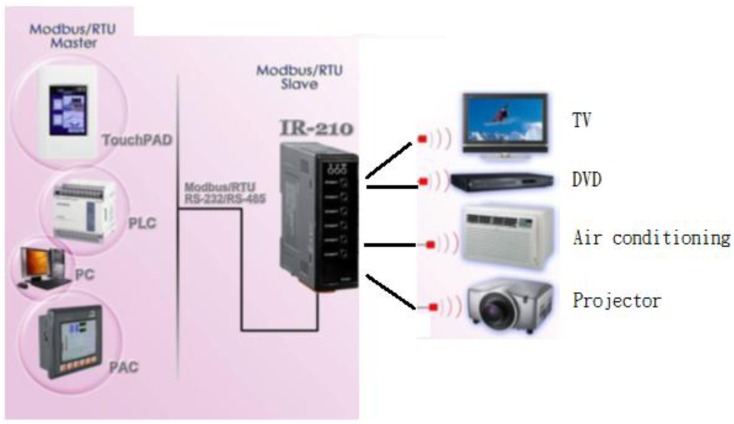
An infrared learning remote module.

An IR learning remote module is made up of an IR Code transmitter and an IR Receive decode control and circuitry involves an oscillation transmitter, a receiver and a codec, as illustrated in Figure 8. Once the remote control encoder is enabled, a selected serial encoded signal is firstly emitted by an IR transmitter, then reflected by a target, received by an IR receiver, and decoded. Provided there is a consistency between the decoded and encoded messages, a light source is switched on/off by a driver. As a universal IR learning remote module, IR-210 is equipped with up to 176 IR remote commands and 6 independent output channels for simultaneous operations at 6 IR carrier frequencies between 32.768 and 56 KHz. RS-232/485 are Modbus RTU interfaces, providing 256 IDs for system extension. IR-210 is a module particularly designed for the application to IR remote control home and office automation, e.g., A/V entertainment, video conference, lighting control, and many more.

### 4.5. Lighting Control Module

**Figure 9.**
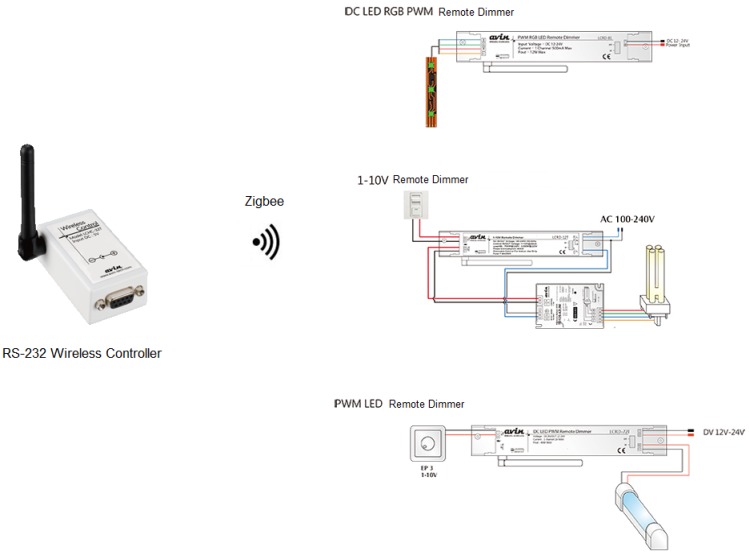
The signal link between a wireless controller and respective remote light dimmers.

**Figure 10.**
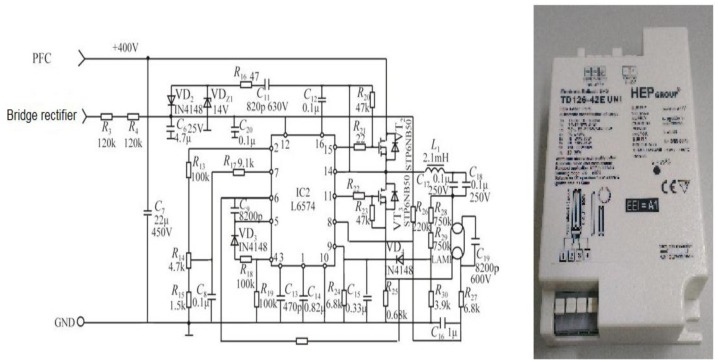
Circuit and photo of an electronic ballast.

This proposal is a ZigBee complinat lighting system. In compliance with IEEE 802.15.4, ZigBee is a short range, low power, communication protocol with a low transmission rate stipulated by the ZigBee alliance. With the Master/Slave features, it provides bidirectional data transmission. There are three frequency bands authorized for ZigBee use, *i.e.*, 2.4 GHz ISM, 915 MHz and 869 MHz bands. It provides a low transmission rate between 10 and 250 Kbps, a narrow band but a low cost transmission protocol. Moreover, ZigBee alliance stipulates a communication protocol for wireless lighting control, and the features thereof are briefly stated as follows.

(A) The operations of light switches, light dimmers and light sensors are stipulated for product compatibility. As illustrated in Figures 9 and 10, the major advantage of a ZigBee network is the network flexibility thereof, that is, a light switch, a dimmer, a remote control, or a light sensor can be employed as either a coordinator or a router.
(B) By use of a ZigBee network coordinator, any controllers or lighting facilities can be added into or removed from a network, a flexibility of a ZigBee-based lighting control network.
(C) The ZigBee technique is expected to dominate the future trend of short range wireless system development, and applications thereof can be extended with ease into smart office or residence automation.

**Figure 11.**
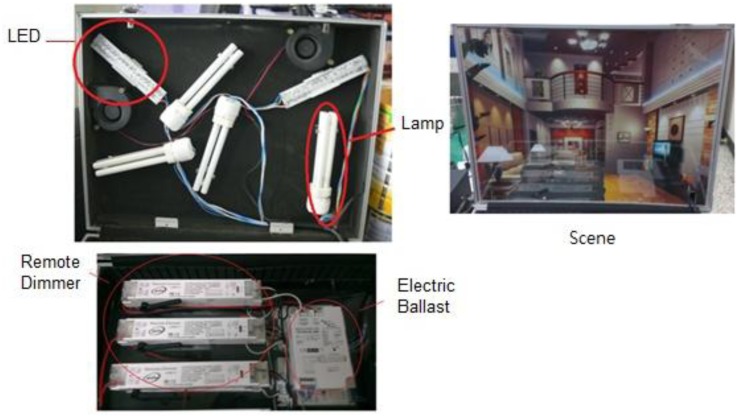
Photos of light sources, remote light dimmers, electronic ballast and scene light field demonstration.

As pictured in Figure 11, a lighting control module is comprised of a remote light dimmer followed by an electric ballast and then by a set of energy saving light bulbs or LEDs. In this context, wireless remote control light sources are provided to meet the user's specific needs, including specified light brightness or light mode control, by a system server via a remote light dimmer. A scene is lit by the remote control light sources placed behind curtains.

The SDI (Signal Digital Interface) for the electronic dimming ballast control signal is Manchester Encoded. As a non-polar signal, it is applicable to data transmission and synchronization. The brightness, in the range of 1% to the specified level particularly for lit scenes in various modes, varies exponentially in exactly the same way as human eyes respond to light. Another point worthy of mention is that electric ballast can be directly switched off by a DSI for energy saving purposes [2].

High performance LED driver ICs are designed to speed up the development of smart LED-based lighting systems. In this context, non-blinking light dimmers and even a remote control lighting system can be implemented so as to meet the color quality requirement and raise the prices of products. The innovative development of LED manufacturing, color and packaging techniques is seen as promising in today's market. A major advantage of LEDs over other types of light sources is the tremendous cost saving and a long lifetime.

LEDs are demonstrated as a revolutionary lighting device in market featuring low power consumption as well as a long term operation. This novel type of light source can be integrated into a long distance network or employed in high valued customized lamps. As a key component of the next generation of light sources, a well designed LED driver, together with optic accessories and heat sink, provides the optimized performance for an LED.

## 5. Development Environment

In this work, the following four pieces of application software are involved in the development of the control interfaces and system programs.

### 5.1. Touch Pad Development Tool

**Figure 12.**
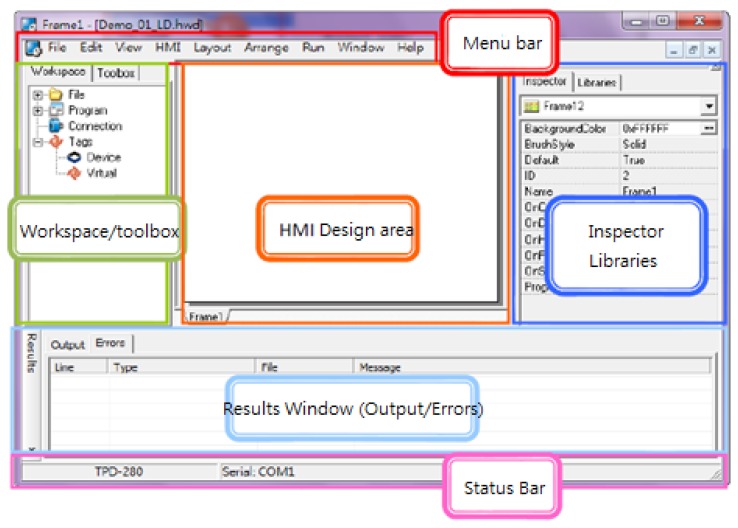
Demonstration of HMIWorks development tool.

As illustrated in Figure 12, HMIWorks is employed as the development tool to design either the ladder diagram or the C programs in Touch Pad.

### 5.2. IR-210 Utility

**Figure 13.**
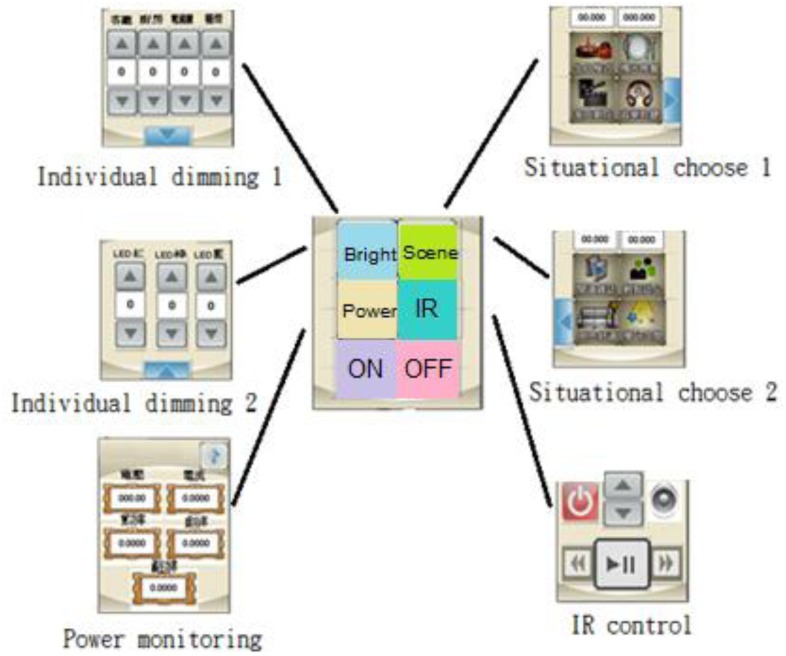
Touch Pad control interface.

**Figure 14.**
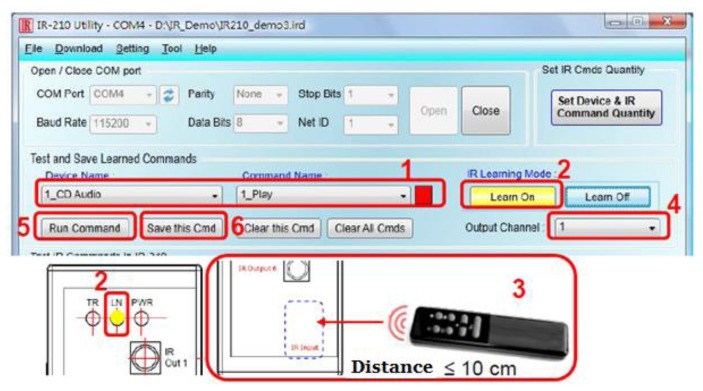
The main window for IR-210 utility.

IR-210 Utility is a Microsoft NET Framework 4 Client Profile based tool for the parameter setting of an IR-210 module and the remote IR learning module. As illustrated in Figure 13, IR-210 is connected to the tool via an RS-232/ RS-485 interface. As presented in Figure 14, there are up to six steps to follow when configuring IR-210, that is, in step 1, enter the name(s) of a controlled device(s) and the command(s); in step 2, click on the Learn On button, and then the LN indicator is lit to signify that the learning mode is enabled; in step 3, the learning items are selected by pointing a remote control at the IR Input button; in step 4, the IR output channel is selected; in step 5, pointing the IR transmitter in the first channel at the controlled device, click on the Run Command button for giving IR commands; in step 6, click on the Save this Cmd button so as to save the learning data. All the devices, as illustrated in Figure 8, can be controlled in exactly the same way.

### 5.3. eLogger

**Figure 15.**
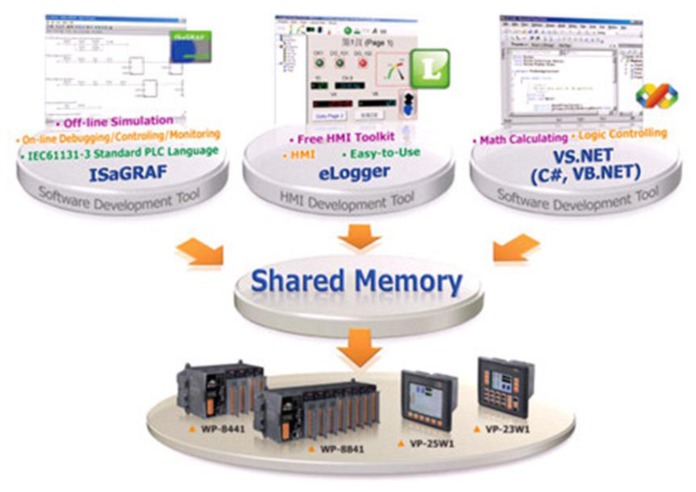
eLogger controllable devices via Shared Memory.

eLogger is a data collection tool to program the human computer interfaces for Windows CE. NET 5.0 based PACs (WinPAC, ViewPAC) and Windows CE .NET 6.0 based PACs (XP-8000 series), such that I/O monitoring and control systems can be constructed in a simple and highly efficient manner. Logic control feature is provided through the Shared Memory, according to which either ISaGRAF or VS.Net or works together with eLogger for the software development for logic controllers. Illustrated in Figure 15 is the way that I/O modules and other register data are controlled through the Shared Memory.

### 5.4. App Inventor

Developed by MIT Media Lab for education purposes, App Inventor is a Scratch-based visual programming language. In this study, it is employed to develop the interfaces for smart phones and tablets. Android, as opposed to iOs, is an open development environment, such that users can develop specific programs to meet their own needs. Besides, App Inventor is a web interface development environment such that programming is made in a visual rather than a conventional way, when developing Android apps, and then can be uploaded to a smart phone directly. Furthermore, online program test can be conducted by App Inventor right after the coding.

## 6. Neural Network-Based Self Adaptive Weighted Data Fusion Approach

**Figure 16.**
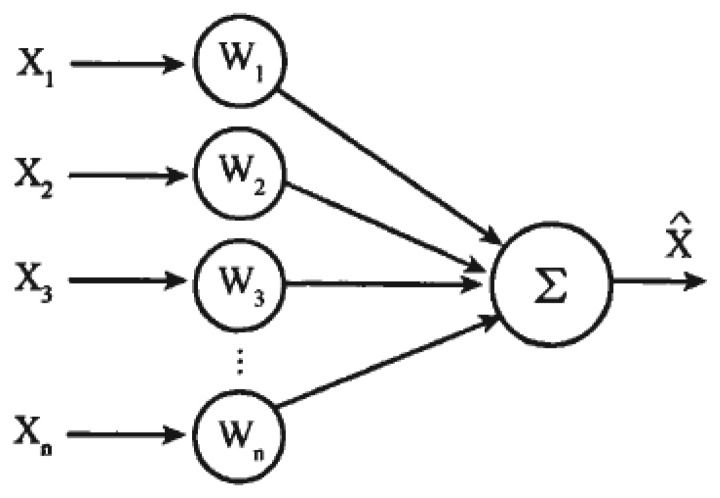
A data fusion model for a self adaptive weighted algorithm.

As illustrated in Figure 16, a so-called self adaptive weighted fusion algorithm refers to an algorithm where respective weights of sensed data are determined in an adaptive manner such that data fusion is optimized [12–15].

Suppose that given an observation area, there are distinct sensed data, e.g., LED 1, LED 2, LED 3, *etc.* As tabulated in Table 1, *P_ij_*,*i*=1,2,3…*r*, *j*=1,2,3…*c*, represent the data sensed about the object *j* by sensor *i*, c the number of the objects that is an unkown, and the variances of data sensed by each node are represented as *σ*_1_,*σ*_2_,.. *σ_r_*:

(3) $$\{{\begin{array}{c} P_{j}=\overset{r}{\underset{i=1}{\text{∑}}}W_{i}P_{ij}\\ \overset{r}{\underset{i=1}{\text{∑}}}W_{i}=1,0\leq W_{i}\leq 1 \end{array}}_{\left(P_{j}\;\text{is the ultimate goal of the}\;j-\text{th observation}\right)}$$

The function $f(w_{1}\ldots W_{r})=\overset{r}{\underset{i=1}{\text{∑}}}W_{i}^{2}\sigma_{i}^{2}$ is minimized as follows:

(4) $$\{\begin{array}{cc} \begin{array}{l} \frac{\partial f}{\partial W_{i}}=0\\ \overset{r}{\underset{i=1}{\text{∑}}}W_{i}=1 \end{array} & ,i=1,2,\ldots r \end{array}$$

the solution is found as:

(5) $$W_{i}=\frac{1}{\sigma_{i}^{2}\overset{r}{\underset{k=1}{\text{∑}}}\frac{1}{\sigma_{k}^{2}}},i=1,2\ldots r$$

and $\frac{\partial }{\partial W_{i}}(\frac{\partial f}{\partial W_{i}})=2\sigma_{i}^{2}>0$. The solutions *W*_1_,…*W_r_* are the weights in this minimization problem:

(6) $$f_{\min}=\frac{1}{\overset{r}{\underset{i-1}{\text{∑}}}\frac{1}{\sigma_{i}^{2}}}$$

and:

(7) $$P_{j}=\frac{\overset{r}{\underset{i=1}{\text{∑}}}\frac{P_{ij}}{\sigma_{i}^{2}}}{\overset{r}{\underset{i=1}{\text{∑}}}\frac{1}{\sigma_{i}^{2}}}$$

It is revealed from Equation (7) that the ultimate goal of the observations *P_j_* shows dependence only on the data sensed by each sensor for a given squared error. Another point worthy of mention is that weighted valuation and valuations in batches can be expressed in exactly the same form, with the only difference that, in the former case, the weightings are assigned to each sensor, while, in the latter, the mean of data is treated as the update, following which the arithmetic mean is defined as:

(8) $$\bar{P}=\frac{1}{r}\overset{r}{\underset{i=1}{\text{∑}}}P_{ij},j=1,2\ldots c$$

and the squared error is: $\sigma_{i}^{2}=\frac{1}{r^{2}}\overset{r}{\underset{i=1}{\text{∑}}}\sigma_{i}^{2}$.

According to Equation (6), the squared error in a neural network based data fusion algorithm is given as $\sigma_{2}^{2}=\frac{1}{\overset{r}{\underset{i=1}{\text{∑}}}\frac{1}{\sigma_{i}^{2}}}$, and according to the Schwartz inequality it is found that:

(9) $$\sigma_{1}^{2}\cdot {\left(\sigma_{2}^{2}\right)}^{-1}=\frac{1}{r^{2}}\sum_{i=1}^{r}\sigma_{i}^{2})\cdot (\sum_{i=1}^{r}\frac{1}{\sigma_{i}^{2}})\geq \frac{1}{r^{2}}{\left(\sum_{i=1}^{r}\sigma_{i}\cdot \frac{1}{\sigma_{i}}\right)}^{2}=1\Rightarrow \sigma_{1}^{2}\geq \sigma_{2}^{2}$$

Thus, it is evident that a low variation in data fusion, *i.e.*, a high stability, is seen in this work.

## 7. Simulation Experiment and Discussion

### 7.1. An Illustration of a Multimeter

**Figure 17.**
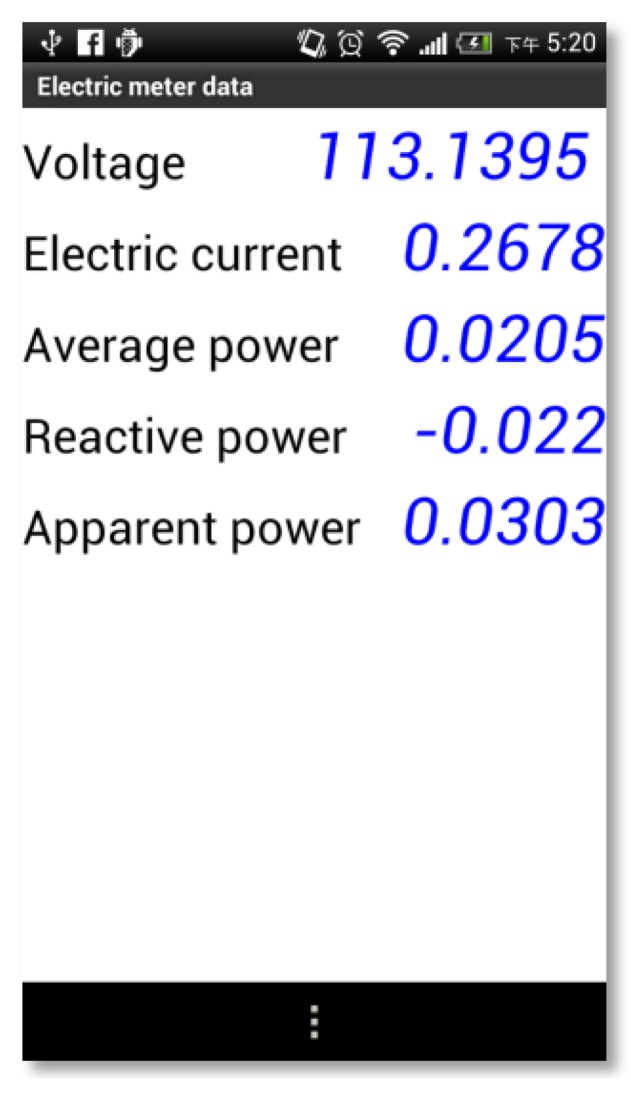
An example of multimeter readings present on a smart phone display.

Demonstrated in Figure 17 is an illustration of reading presentation of a CT on a smart phone, including the voltage, current, average, reactive and apparent power. The readings can be displayed as well on a Touch Pad and on the screen of an XP-8000 controller.

### 7.2. Lighting Mode Demonstration

Preset lighting modes can be easily enabled in a smart lighting system by means of timers and sensors. A clear disadvantage is that any scene change necessitates a change in the lighting mode. As a rule, preset lighting mode control is mainly applied to lobbies, exhibition halls, conference centers, and so forth for various purposes. It can be operated manually or by a central control unit. In the former, a low cost lighting system can be merely switched on/off manually or is simply controlled by a light dimmer, while in the latter; a complicated lighting system must be made centrally controlled such as for large scale performance, either indoor or outdoor. Yet, a centrally controlled lighting system is employed for business use in hotels. Over recent years, home automation, including electric curtain, lighting, A/V systems, emergency call system control, *etc.* is achieved by a combination of a central control unit and a manual controller. In short, a smart lighting system involves multiple types of controllers and control strategies to meet a wide diversity of needs [2].

**Figure 18.**
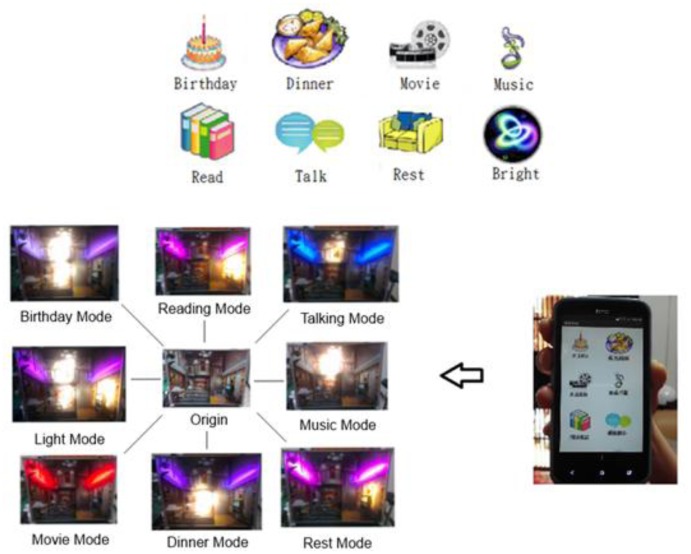
Lighting mode demonstration.

**Figure 19.**
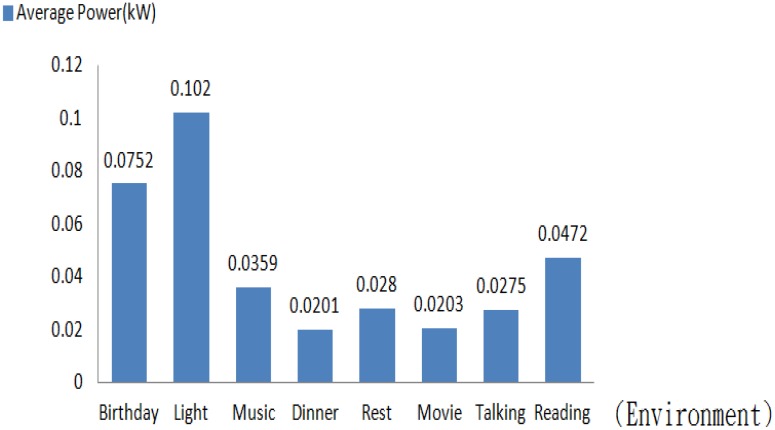
A bar diagram of the average power consumption *versus* the lighting mode.

Illustrated in Figure 18 is a demonstration of a lighting control interface. Each of the preset modes is demonstrated following the user's command. Exhibited in Figure 19 is a bar graph of the average power consumption *versus* the lighting mode. As such, high power consumption is reflected by a high brightness.

### 7.3. LED Light Tuning

**Figure 20.**
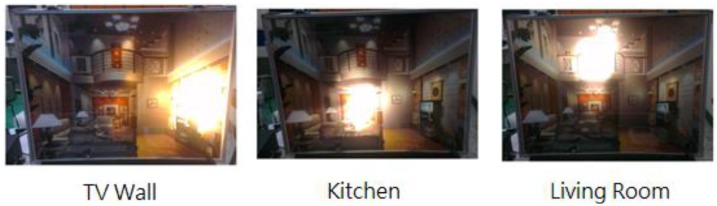
Lighting mode demonstration.

**Figure 21.**
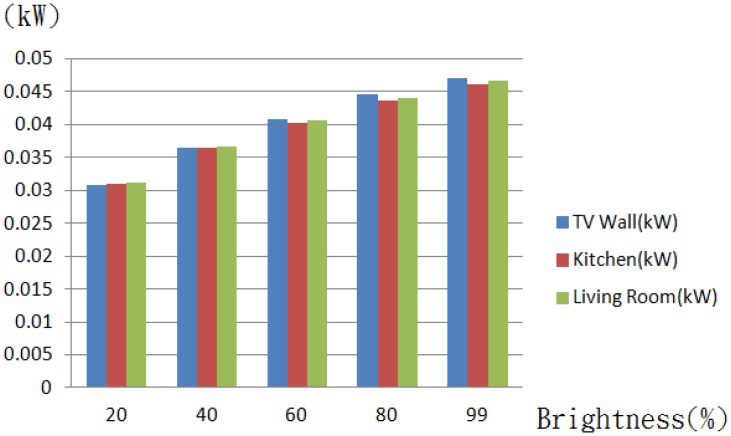
A bar diagram of the average power consumption *versus* the brightness.

The brightness of a dimmable light tube is rated on a scale of 0 to 99. Demonstrated in Figure 20 are presentations of respective lighting modes, and in Figure 21 is a comparison result of the average power consumption *versus* brightness, and a high consistence is seen among the cases of a TV wall, a kitchen and a living room, due to the use of the same model light tubes.

### 7.4. Color Tunable LED

The light of an LED, a color tunable and energy saving light source, is able to cover the entire visible light spectrum. Unlike conventional light sources, it does not require any color filter to filter out unwanted chromatic components. Besides, an LED provides a fast response to input signals and outputs full color promptly. The chromatic and other characteristics of an LED are well maintained during the brightness tuning process, superiority over other light sources, such as neon light, *etc.* The color of light can be decomposed into RGB components, each of which is quantified by as many as 255 levels. For instance, an identical value in RGB components leads to gray color, 255 leads to white, and 0 leads to black.

All the lights can be decomposed into three monochromatic components, each of which cannot be made as a mixture of the others. There is more than one way to choose three basic colors. Stipulated by CIE in 1931, the wavelengths of red (R), green (G) and blue (B) are specified at 700, 541.6 and 435.8 nm, respectively. In such RGB system, equal energy white light is a mixture of RGB according to the following flux ratio:

(10) $$F_{R}:F_{G}:F_{B}=1:4.5907:0.0601$$

In this context, a mixture of 1 lm red, 4.5907 lm and 0601 lm blue light leads to 5.6508 lm white light. As a rule, RGB are referred to as the amount of color. The flux at a certain wavelength F is represented as:

(11) F=R(R)+G(G)+B(B)

The color of light can be alternatively determined by the chromaticity coordinates, the relative values of RGB, namely. r, g, b, defined respectively as:

(12) $$\begin{array}{c} r=\frac{R}{R+G+B}\\ g=\frac{G}{R+G+B}\\ b=\frac{B}{R+G+B} \end{array}$$

Tabulated in Table 2 are the RGB color specifications of a typical high power LED.

**Figure 22.**
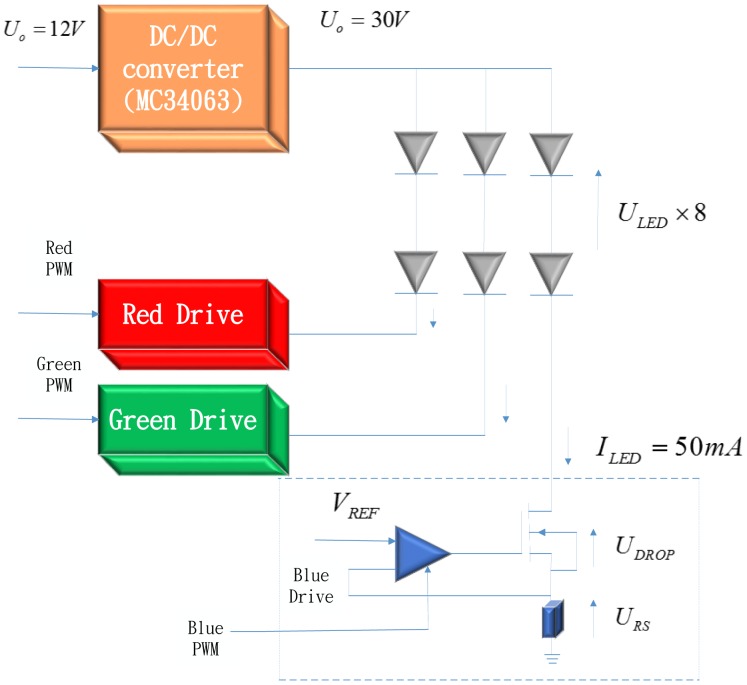
RGB LED PWM driver modules [2].

**Figure 23.**
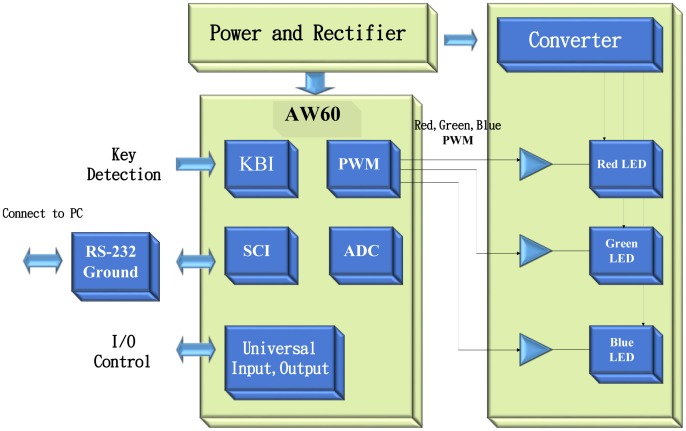
RGB control module for an LED [2].

Exhibited in Figure 22 is a RGB PWM LED driver, while instead in Figure 23 is a micro controller based PWM driver [2].

In a complete closed loop color control system, color compensation must be made to yield the intended chromatic light. The consistency in light output can be well maintained by either linear or PWM constant current LED drivers. The choice is made considering multiple quantities of interest, e.g., the efficiency, input voltage range, the number of LEDs, *etc.* There are plenty of ways to control all the drivers’ output current. To begin with, a reference voltage can be generated by either a DAC or a digital potentialmeter in such a way that the output current swing can be made up to the rated output current. Besides, PWM signals are provided by MCU as a way to modulate the driver's output current. A PWM controller must be operated at high switching frequency to avoid any flashing light.

**Figure 24.**
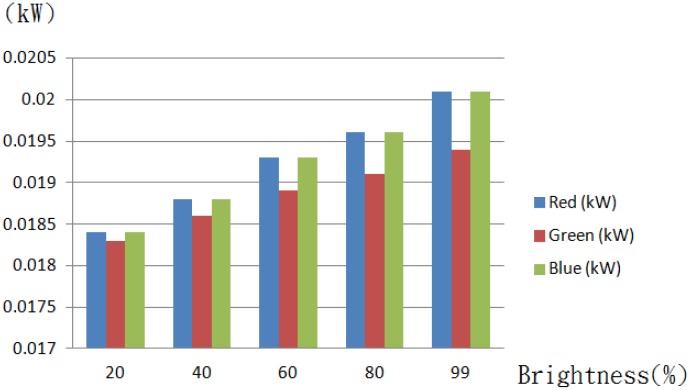
A bar diagram of the average power consumption in RGB components *versus* the brightness.

The above experiment indicates a high power consistency among each of the RGB components for identical brightness. As illustrated in Figure 24, a high consistency is seen as well for various combinations of RGB components, namely R + G, G + B, G + B and R + G + B.

## 7.5. Analysis on LED Temperature and Luminance

**Figure 25.**
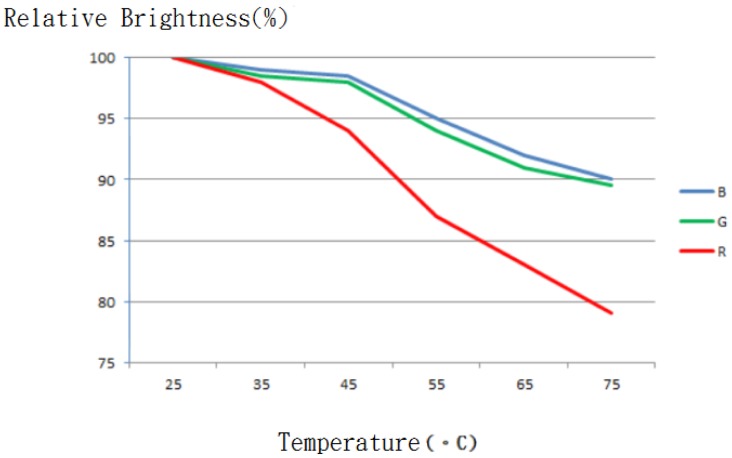
A plot of relative luminance *versus* LED temperature.

In an attempt to reach a thermal equilibrium state, this experiment is conducted an hour after the system is powered on for the investigation into heat effect on the LED color. As illustrated in Figure 25, there is a more rapid relatively luminance descent in R than in G and B color components, as the operating temperature rises. For this sake, the RGB components must be compensated so as to regulate the LED color. In this work, this is done by the minimization of the squared error through a self adaptive weighted data fusion algorithm. An alternative way to deal with the problem is to put an effort directly into the issue of LED cooling, a key area of our future research project. LEDs receive global attention due to its color tenability, but a disadvantage gained is the time drift characteristic, leading to a need to develop an MCU based LED driver involving light sensors to regulate the LED color and elevate the efficiency accordingly.

### 7.6. Performance Comparison

**Figure 26.**
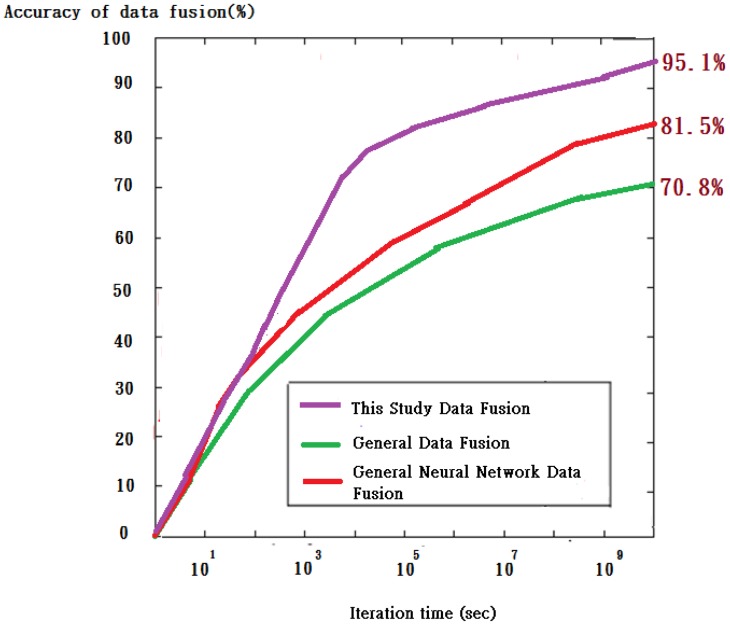
A comparison result of the relative acceptance rate *versus* the estimated time between theoretical approaches and experiment.

In Figure 26, as the estimated time approaches 10^9^ s, the acceptance rate tends to match toward the end of an observation period among the measurement, referenced from a piece of our prior work [16], and two theoretical approaches, *i.e.*, an optimization-based data fusion approach and a standard parallel data fusion approach. In terms of training errors, the optimization-based approach is found experimentally to be superior to the others. A superior performance is seen in a self adaptive weighted approach than in a mean value approach, unless an identical squared error is shared by all the sensors.

Yet, it is a mission impossible in practical applications due to the fact that external disturbance is a time varying uncertain function. The squared error in each sensor is assessed by multiple sensed data sources, but real time evaluation cannot be performed just as encountered in a recursive estimation of single sensor. For this sake, a batch process strategy is employed for a given squared error to perform data fusion according to Equation (7). In this work, the self adaptive weighted approach is found to be the best in terms of data fusion performance, while the mean value approach is the worst instead. Although this proposed algorithm is validated as a way to significantly elevate both the efficiency and precision, the computational complexity will be raised due to a large number of sensors. Hence, there is a trade-off between the precision and efficiency in practical applications [17].

**Figure 27.**
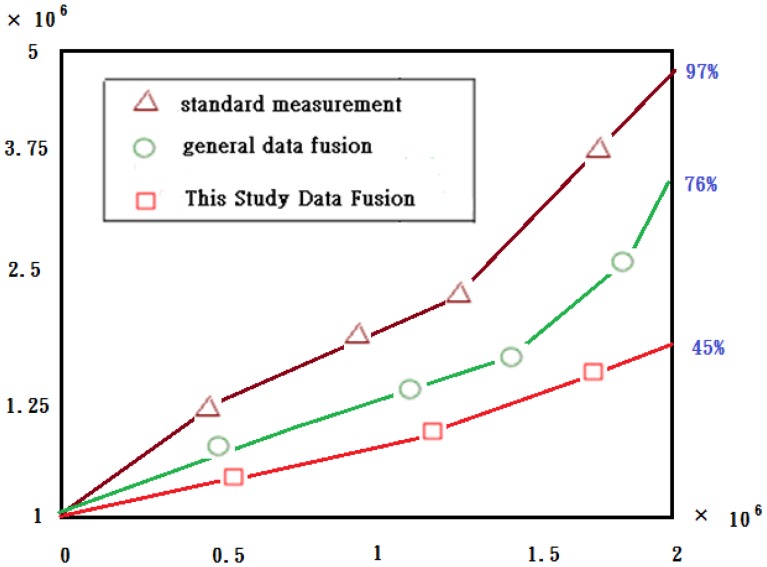
Graphical comparison of computational complexity for the various data fusion methods.

Figure 27 illustrates the results from the computational complexity derivations for the various data fusion. From Figure 27 it is evident that the overhead incurred by the multirate data fusion is negligible compared to the overall savings in the time domain case. The self-adaptive weighted approach shows the expected 21% reduction over the general data fusion. It is also very evident that this study has excellent computational complexity efficiency between the general data fusion and standard multirate data fusion implementation measurement [18,19].

## 8. Conclusions and Future Work

Remote control for home appliances can be operated by a handheld device, such as a smart phone, a tablet, *etc.* The performance superiority of an LED lighting system over its conventional counterparts is demonstrated in the proposal in many aspects. Further cost savings are realized by the proposed color-changing and fully dimmable smart LED lighting controller relative to a conventional one.

This work is validated as a very effective approach to the performance improvement of an LED lighting system under various circumstances such as art exhibitions, alarm indicators in plants for safety concern, and many more. This smart LED lighting system prevents light flashing problem for the sake of vision health. The brightness can be precisely controlled to meet the user's need at a specific place and time. Besides, it serves as a voltage regulator as well to prevent LED damage from the voltage over and undershoot, that is, the lifetime of an LED light source is hence extended.

A program starts technique is adopted in a smart light dimmer such that a specified level of brightness is reached over time for an extended lifetime of an LED. Likewise, a program close technique is employed as well. It is found that a 10% drop in brightness can double the lifespan of an LED light source, while a 50% drop can extended the lifespan by up to 20-fold. Preset lighting modes are stored in the memory of a micro controller unit for easy maintenance as well as replacement. A number of automated services are expected to be provided to meet specific needs and for energy saving purposes. In short, a tremendous amount of advancing progress has been made in the field of smart lighting techniques, as the consequence of network and automatic control technique improvement.

## Figures and Tables

**Table 1.** Sensed data nomenclature.

The first sensor	P_11_	P_12_	……
The second sensor	P_21_	P_22_	……
……	……	……	……
The R-th sensor	P_r1_	P_r2_	……

**Table 2.** Specifications of a high power RGB LED.

**Items**	**Dominant** **Wavelength/nm**	**Forward** **Current/mA**	**Forward** **voltage/V**	**Flux/lm**	**Chroma x**	**Chroma y**

**Color**						
R	625	350	2.77	39.6	0.700	0.299
G	537	350	3.49	41.6	0.223	0.714
B	458	350	3.76	6.5	0.149	0.031
